# Chest CT Findings after Mild COVID-19 Do Not Explain Persisting Respiratory Symptoms: An Explanatory Study

**DOI:** 10.3390/diagnostics13091616

**Published:** 2023-05-03

**Authors:** Stefan Malesevic, Noriane A. Sievi, Jonas Herth, Felix Schmidt, Dörthe Schmidt, Florence Vallelian, Ilijas Jelcic, Lisa Jungblut, Thomas Frauenfelder, Malcolm Kohler, Katharina Martini, Christian F. Clarenbach

**Affiliations:** 1 Faculty of Medicine, University of Zurich, 8006 Zurich, Switzerland; 2 Department of Pulmonology, University Hospital Zurich, 8091 Zurich, Switzerland; noriane.sievi@usz.ch; 3 Department of Cardiology, University Hospital Zurich, 8091 Zurich, Switzerland; 4 Department of Internal Medicine, University Hospital Zurich, 8091 Zurich, Switzerland; 5 Department of Neurology, University Hospital Zurich, 8091 Zurich, Switzerland; 6 Institute of Diagnostic and Interventional Radiology, University Hospital Zurich, 8091 Zurich, Switzerland

**Keywords:** post-COVID-19, chest CT, respiratory symptoms

## Abstract

(1) Background: Lung tissue involvement is frequently observed in acute COVID-19. However, it is unclear whether CT findings at follow-up are associated with persisting respiratory symptoms after initial mild or moderate infection. (2) Methods: Chest CTs of patients with persisting respiratory symptoms referred to the post-COVID-19 outpatient clinic were reassessed for parenchymal changes, and their potential association was evaluated. (3) Results: A total of 53 patients (31 female) with a mean (SD) age of 46 (13) years were included, of whom 89% had mild COVID-19. Median (quartiles) time from infection to CT was 139 (86, 189) days. Respiratory symptoms were dyspnea (79%), cough (42%), and thoracic pain (64%). Furthermore, 30 of 53 CTs showed very discrete and two CTs showed medium parenchymal abnormalities. No severe findings were observed. Mosaic attenuation (40%), ground glass opacity (2%), and fibrotic-like changes (25%) were recorded. No evidence for an association between persisting respiratory symptoms and chest CT findings was found. (4) Conclusions: More than half of the patients with initially mild or moderate infection showed findings on chest CT at follow-up. Respiratory symptoms, however, were not related to any chest CT finding. We, therefore, do not suggest routine chest CT follow-up in this patient group if no other indications are given.

## 1. Introduction

The novel coronavirus, SARS-CoV-2, has caused the most recent deadly pandemic. Patients and caregivers are dealing with the unclear long-term impact of the infection. Lower-respiratory-tract involvement is frequently observed in the acute phase of infection. On chest computed tomography (CT) scans during the acute stage of COVID-19, patients with a severe course of the disease typically present with progressive ground glass opacities (GGOs) and consolidations mainly distributed in the peripheral lung, along with rounded opacities and a crazy-paving pattern [1]. Several studies showed that patients with severe acute infection had remaining radiological abnormalities at follow-up CT [2,3,4,5]. In particular, the development of acute respiratory distress syndrome (ARDS) during initial infection was associated with the formation of fibrotic-like changes in CT images [6]. In addition to interstitial changes and consolidations, pneumatoceles and air trapping are relevant findings in the follow-up of these patients [3,5]. Although the mechanisms leading to the development of pulmonary fibrosis through viral infections are not fully understood, direct lung damage or overactivation of the immune system with the release of a large number of proinflammatory and profibrotic cytokines might play a key role [7]. These changes on imaging are often accompanied by impaired diffusion capacity or restrictive ventilation disorder in lung function testing.

Interestingly, patients who initially suffered from mild or moderate symptoms from a SARS-CoV-2 infection without the need for hospitalization are also capable of developing symptoms that can last months after acute disease [8,9] and substantially affect health-related quality of life [10]. Long-term involvements including thoracic pain, dyspnea, or coughing are frequently reported in patients with only mild to moderate symptoms during the acute phase of the disease [8,11,12]. Researchers are still trying to unravel the mechanisms leading to the long-term health consequences termed “post-COVID-19 syndrome”. So far, persistent inflammation, induced autoimmunity, or viral persistence have been suggested as potential drivers [13].

To the best of our knowledge, it has not been thoroughly investigated if patients suffering from post-COVID-19 symptoms with persisting respiratory symptoms after a mild or moderate acute infection could also have developed persisting alterations of the lung parenchyma associated with ongoing respiratory symptoms. Our clinical experience showed that chest CT scans have only rarely been performed in this patient group, without any structured examination. We, therefore, assessed chest CT scans of patients with post-COVID-19 syndrome after a mild or moderate acute infection to investigate if there were objective long-term damages on lung parenchyma and evaluated the association with persisting respiratory symptoms.

## 2. Materials and Methods

### 2.1. Study Design and Patient Population

The University Hospital Zurich established a post-COVID-19 outpatient clinic for patients with post-COVID-19 syndrome, involving the departments of pulmonology, neurology, cardiology, and internal medicine. Included were patients who visited this outpatient clinic, suffered from ongoing symptoms after an acute mild or moderate SARS-CoV-2 infection, and had a chest CT scan performed at least 1 month after acute infection. Chest CT scans were mostly performed to exclude relevant structural lung damages without previous clinical findings (e.g., impaired lung function or low oxygen saturation). Patients with an initial severe course of COVID-19 disease that required prolonged hospitalization or intensive care treatment, as well as patients with an alternative diagnosis for reported symptoms were excluded. The study was conducted in accordance with the Declaration of Helsinki, and all subjects provided written informed general consent. The Ethics Committee of the Canton of Zurich approved the study (BASEC 2021-00280), and the study was registered on www.ClinicalTrials.gov (NCT04793269, accessed on 2 May 2023).

### 2.2. Symptoms and Clinical Parameters

Demographics (sex, age, BMI, and smoking status), symptoms during acute infection, and post-COVID-19 symptoms was extracted from systematically documented medical reports. Symptoms were only coded as absent if recorded in the medical record as “absent”. Dyspnea was additionally graded according to severity by the Modified Medical Research Council Dyspnea Scale (mMRC). Chest CT scan indication was evaluated if patients showed abnormal clinical findings. In patients with persisting respiratory symptoms and no relevant abnormal clinical findings, pro and cons of chest CT scan for increasing the certainty for exclusion of relevant lung damages were individually discussed, and chest CT scan was performed if requested. Comorbidities that were pre-existing before acute COVID-19 infection were recorded. Furthermore, standard pulmonary functional testing according to ATS/ERS guidelines was performed (forced expiratory volume in 1 s (FEV1), forced vital capacity (FVC), diffusion capacity of the lung for monoxide (TLco), and carbon monoxide transfer factor (Kco)).

### 2.3. CT Image Acquisition

Single-energy CT was performed in all patients on different CT scanners (NAEOTOM Alpha, SOMATOM Force, SOMATOM Definition AS, SOMATOM Edge Plus, Cardiac64 or SOMATOM Definition Flash; Siemens Healthcare, Forchheim, Germany and Brilliance 16P, Philips, Amsterdam, The Netherlands) with or without the application of an intravenous contrast agent in supine position at full inspiration. No specific post-COVID-19 protocol was applied. The protocol depended on the referral question. In a subset of patients where expiratory CT in prone position was available, the scans were additionally analyzed. CT scans were performed at 80 to 120 kVp with varying tube currents and a pitch of 1–1.2. Images were reconstructed at a slice thickness of 0.75–2 mm using iterative reconstruction algorithms.

### 2.4. Systematic Evaluation of CT Scans

Two radiologists with long-term experience in thoracic imaging, blinded to patient symptoms, evaluated the images for the presence of parenchymal changes according to the glossary proposed by the European Society of Radiology (ESTI) [5]; presence (yes/no) and severity (discrete, medium, or strong) of parenchymal changes such as GGOs, fibrotic-like changes (i.e., reticulation, bronchial dilatation, subpleural bands, and mediastinal and hilar lymphadenopathy), honey combing, and mosaic attenuation as signs of air trapping were assessed on expiratory CT scans. In cases with no agreement, a third blinded radiologist was consulted for definite judgement. If no expiratory CT was available, minimum intensity projection (MinIP) reconstructions were used to detect mosaic attenuation as a sign of air trapping.

### 2.5. Statistical Analysis

Descriptive statistics of baseline patient characteristics were presented as the mean and standard deviation (SD) or median and quartiles (25% and 75% quartiles) for continuous measurements, and as the number and percentage of total for categorical measurements. Multivariable logistic regression analyses adjusted for relevant confounders for symptoms (pre-existing asthma for thoracic pain, dyspnea, and cough; smoking and BMI for dyspnea) were performed to evaluate chest CT scan findings as possible predictors for persisting respiratory symptoms. The association of dyspnea severity (mMRC) with chest CT scan findings was analyzed by ordinal logistic regression, adjusted for age, pre-existing asthma, smoking history, and BMI. The association between dyspnea and lung function was tested using a logistic regression and adjusted for pre-existing asthma, smoking, and BMI. Inter-rater reliability was calculated using Cohen’s kappa coefficients and categorized as poor (k < 0.20), fair (k = 0.20–0.40), moderate (k = 0.41–0.60), good (k = 0.61–0.80), or very good (k = 0.81–1.00) [14]. No a priori sample size calculation was performed due to the explanatory study design. All statistical tests were two-tailed, and a *p*-value of <0.05 was considered statistically significant. Statistical analysis was performed using Stata version 16.1 (StataCorp. 2019, Texas, TX, USA).

## 3. Results

### 3.1. Patient Population

In total, 396 patients were seen in the post-COVID-19 ambulatory between February 2020 and March 2022. Chest CT was performed in 53 patients. In seven patients, chest CT was performed due to abnormal clinical findings (abnormal lung function, enhanced D-dimer, suspicious chest X-ray, lung embolism during acute COVID-19, and discrete crackling sound of the lung), whereas 46 patients requested chest CT scan for a definite exclusion of relevant lung damages. A total of 31 patients (58.0%) were female. The mean (SD) age of patients was 45.9 (13.1) years, and the median (quartiles) BMI was 25 (21.8, 27.7) kg/m^2^. Most patients (89.2%) had an initial mild infection according to the World Health Organization (WHO). The median (quartiles) times from symptom onset of acute infections and from first outpatient clinic visit to performing the chest CT scan were 139 (86, 189) days and 14 (3, 37) days, respectively. An indication for chest CT scan was persisting respiratory symptoms. Only eight (15.0%) patients were current smokers, and other respiratory diseases were scarce (seven patients with pre-existing asthma; one patient with pre-existing COPD).

### 3.2. Symptoms and Clinical Parameters

Most patients showed normal lung function and diffusion capacities with mean (SD) predicted FEV1 of 89.9% (12.8%) and mean (SD) predicted Kco of 91.3% (18.7%). All patient characteristics are outlined in Table 1.

At acute infection, 85.7% of the patients showed dyspnea, 89.1% had a cough, and 81.8% suffered from thoracic pain. The frequency of current respiratory symptoms was as follows: 42 (79.0%) patients with dyspnea, 22 (42.0%) patients with cough, and 34 (64.1%) patients with thoracic pain. Current symptom frequencies are shown in Table S1 (Supplementary Materials).

### 3.3. Chest CT Scan Findings

In total, 17 out of 53 CT scans (32%) were performed with intravenous contrast agent. In 56.6% of cases (N = 30), an expiratory CT scan in prone position was also available. Discrete lung abnormalities were found in 30 (56.6%) and medium lung abnormalities were found in two (6.9%) of 53 chest CT scans with possible post-COVID-19 features in 29 (54.7%) patients. No patient showed severe chest CT scan findings. The findings are outlined in Table 2.

**Figure 1:**
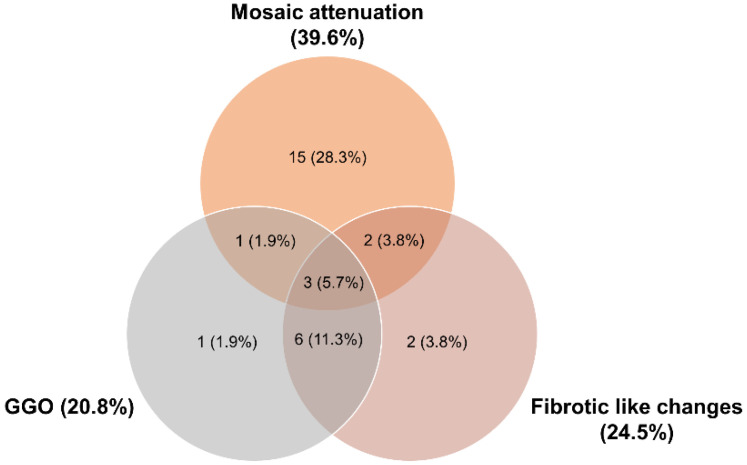
Prevalence of the most dominant chest CT findings (mosaic attenuation, ground glass opacities (GGOs), and fibrotic-like changes) in a Venn diagram.

Most commonly, mosaic attenuation (39.6%), ground glass opacities (GGOs) (20.8%), and fibrotic-like changes (24.5%) were recorded. Mosaic attenuations mostly appeared isolated, whereas GGOs and fibrotic-like changes often occurred in combination. Rarely, all three findings occurred simultaneously (Figure 1). Honey combing was reported in one case. Inter-rater reliability was categorized as “very good” for all addressed findings (Table 3).

**Figure 2:**
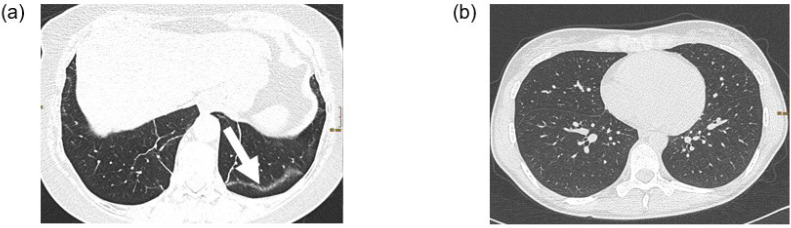
Two examples of chest CT scans. (**a**) CT examination in supine position of a 62 year old female patient with post-COVID-19 syndrome. The lung parenchyma shows subpleural bands (arrow) in the left lower lobe as sign of residual post-COVID changes. (**b**) CT examination in prone position of a 32 year old female patient with post-COVID-19 syndrome. The lung parenchyma is unremarkable.

Figure 2 shows an example of a patient with possible post-COVID-19 features (subpleural bands) and a patient with a normal chest CT scan.

### 3.4. Correlation of Clinical Parameters and Chest CT Scan Findings

Multivariable logistic regression analyses showed no evidence of an association between the most common radiological chest CT findings and persisting respiratory symptoms such as thoracic pain, dyspnea, and cough. Lung function or diffusion capacity was not associated with dyspnea (Table 4). No associations between severity of dyspnea (mMRC) and the various chest CT scan findings were found.

## 4. Discussion

This study investigated the association of persisting respiratory symptoms with the presence of parenchymal changes on chest CT scans in post-COVID-19 patients who initially suffered from a mild or moderate COVID-19 infection. Although more than half of the patients showed findings on imaging that might be attributed to post-COVID-19 features, the majority of chest CT findings were only subtle and discrete, mostly in the subpleural parts of the lung, and the reported respiratory symptoms were not related to the chest CT abnormalities.

Various studies investigated chest CT scans of asymptomatic COVID-19 patients during the acute phase of the disease. A systematic review and meta-analysis included seven studies with 231 asymptomatic COVID-19 patients during acute infection. The pooled (95% CI) estimate of positive chest CT findings (e.g., GGOs, stripe shadows, and interlobular septal thickening) in patients that remained asymptomatic despite infection was 62% (38, 81) [15]. A study by Uysal et al. [16] investigated 64 asymptomatic COVID-19 patients during acute infection (mean (SD) age of 59.6 (12.3) years, 65% female) with no abnormal clinical and laboratory findings and found lung involvements in 75% of the participants. GGOs were the most common finding in 63% of the individuals. Furthermore, chest CT scans of recovering patients after the SARS-CoV-1 pandemic in 2003 showed persistent GGOs and reticular opacities with mild traction bronchiectasis resembling fibrotic-like changes in the lung [17].

A proportion of patients with initial mild or moderate COVID-19 infection show remaining respiratory symptoms such as dyspnea or thoracic pain. To date, the mechanisms leading to these prolonged symptoms are still unknown, and usual diagnostic tests in clinical routine show no relevant abnormal finding. Long-term changes in chest CT scans have been thoroughly evaluated in patients who initially suffered from a severe infection that required hospitalization and, in some cases, even intensive care treatment [5,18,19]. Chest CT abnormalities resolved 3 months after acute infection in 61% of patients and 12 months after acute infection in 75% of patients [19]. In patients with remaining abnormalities, these changes are associated with reduced diffusing capacity [20]; rarely, persistent lung damage can translate into irreversible fibrotic changes. However, chest CT scans of patients with ongoing post-COVID-19 symptoms after a mild or moderate infection have not been thoroughly investigated. One study investigated chest CT scans of eight patients on average 4 months after an initial mild or moderate COVID-19 disease and found a hypoattenuation mosaic pattern (13%), reticulations (13%), and architectural distortion (13%) [21]. Zhou et al. [1] assessed the sequelae of 120 mostly mild COVID-19 patients 1 year after acute infection. In the non-severe cases, 47 of 83 (56.6%) chest CTs showed abnormal findings at 12 month follow-up with 13.3% GGOs and 16.9% fibrotic-like changes. Although two-thirds of these mild to moderate cases showed no persisting symptoms in contrast to our study population with ongoing post-COVID-19 symptoms, the prevalence of GGOs was only slightly higher (13.3% vs. 20.8%). The prevalence of fibrosis like pattern (16.9%) was slightly lower than the 24.5% fibrotic-like changes found in our population. However, chest CT scans were performed a median of 4.5 months after acute infection in our patient sample compared to 12 months in the study above, and studies with severe COVID-19 patients showed that parenchymal changes are likely to disappear within 12 months of recovery [5]. Mosaic attenuation is a nonspecific finding that can be associated with small airway diseases, vascular causes, or parenchymal diseases. Often, it results from air being trapped in some regions of the lung following expiration. However, mosaic attenuation and air trapping can also be seen in healthy individuals with normal lung function testing and, therefore, do not have a pathologic value in all cases [22,23]. In a previous study, 21% of chest CT scan readings of healthy individuals (median age of 44 years, 64% female) showed mosaic lung attenuation [19]. Therefore, it is important to differentiate significant mosaic attenuation from minor physiologic variations in attenuation that involve fewer than four secondary pulmonary lobules since this appearance has been observed with equal frequency in asthmatics and healthy subjects [24]. In our study population, discrete mosaic attenuation was found in 39.6% of the patients with no moderate or severe radiologic distribution. Overall, we cannot conclude if these lung abnormalities are indeed being caused by the SARS-CoV-2 infection or pre-existing since no pre-infection chest CT scans were available in our population. Regardless of whether the chest CT scan findings were caused by the SARS-CoV-2 infection, persisting respiratory symptoms such as dyspnea, cough, and thoracic pain were not associated with the chest CT findings or lung function testing. These findings support the hesitant investigation of CT imaging in patients with ongoing symptoms after a mild or moderate COVID-19 infection with no abnormalities in lung function testing or oxygen saturation. Therefore, further radiological investigation in these patients seems unnecessary, unethical due to radiation exposure, and uneconomical. Although the findings have to be confirmed in a larger, prospective study, the results from our study seem to have important clinical implications and should guide clinicians in follow-up of post-COVID-19 patients after a mild or moderate acute infection. The results do not suggest to routinely perform chest CT scans in this patient population, as the probability of discovering abnormalities within 6 months after acute infection is relatively high, but the findings are mostly discrete and no evidence for an association with persisting respiratory symptoms was found. Furthermore, considering that the course after severe infection shows mostly resolving parenchymal alteration after 12 months [19], we assume that this positive evolution is at least comparable in milder cases. We recommend that chest CTs should only be performed if there are conspicuous features during clinical examination such as hypoxemia, if lung function testing is suggestive of obstructive or restrictive lung disease, or if other indications from the clinicians ask for an additional imaging modality.

A research group performed a pilot study with hyperpolarized Xenon MRIs in 11 non-hospitalized patients with ongoing dyspnea but normal chest CT scans, and then compared them to 12 hospitalized COVID-19 patients. Both groups showed comparable changes on MRI imaging; therefore, they suggested that long-lasting microstructural abnormalities due to SARS-CoV-2 infection might be responsible for these abnormalities in both groups [25]. Since the study was not powered for statistical analysis, the findings have to be confirmed in a larger study sample. However, we cannot exclude that the persisting symptoms in our post-COVID-19 cohort could be explained by lung abnormalities not seen on chest CT scans, and that alternative radiological techniques would be able to detect relevant pathologies. Furthermore, we cannot exclude that the persisting symptoms are related to other functional impairments that were not investigated as part of this study. As respiratory symptoms such as dyspnea, thoracic pain, or cough are very unspecific symptoms with various causes (e.g., cardiac and psychiatric), causes other than structural pathologies of the lung for persisting symptoms have to be investigated.

Our study had some limitations. None of our patients had a chest CT scan at the time of the acute SARS-CoV-2 infection or before the SARS-CoV-2 infection. Therefore, we cannot conclude if the changes seen on imaging occurred as a result of the SARS-CoV-2 infections or if these were present even before the infection. Nonetheless, the chest CT findings are not associated with any respiratory symptoms occurring after SARS-CoV-2 infection; therefore, the presence of a pre-existing chest CT was not relevant according to our research question. Second, homogeneity was missing with regard to the timepoint of the chest CT scan relative to the acute infection (ranging from 30 days to 530 days). However, this could also be interpreted as the timepoint of imaging not having an impact on our outcome. Due to the explanatory design, no a priori sample size calculation was performed. Therefore, the sample size was possibly too small to show statistical significance, and the results have to be confirmed in lager, multicenter studies. Third, in an optimal and prospective setting, only HRCT examinations without intravenous contrast agent, with 1 mm reconstructions in expiratory acquisition, should have been used. Unfortunately, in our retrospective study, chest CTs were performed on scanners from different vendors, using different acquisition and reconstruction parameters. Furthermore, the referring clinicians were often asked to rule out pulmonary embolism in the post-COVID-19 setting. Therefore, some CTs were performed with intravenous contrast agent, potentially hampering the evaluation of pulmonary parenchyma. Nevertheless, the relatively young patient cohort would have precluded the need to scan the patients twice (one without and once with contrast agent) for evident reasons.

## 5. Conclusions

Chest CT scans of post-COVID-19 patients who initially suffered from a mild or moderate COVID-19 infection showed discrete findings in more than half of the patients 5 months after acute infection. Persisting respiratory symptoms, however, were not related to any chest CT findings. Routine chest CT follow-up in this patient cohort seems not to be helpful for regular clinical workup if no other indications are given.

## Figures and Tables

**Table 1:** Patient characteristics.

N	53
**Sex, N (%)**	
Female	31 (58.0)
Male	22 (42.0)
**Age, mean (SD)**	45.9 (13.1)
**BMI kg/m^2^, median (IQR)**	25.0 (21.8, 27.7)
**WHO classification, N (%)**	
Mild	47 (89.0)
Moderate	6 (11.0)
**Days from first symptoms to CT, median (IQR)**	139 (86, 189)
**Smoking history, N (%)**	
Current	8 (15.0)
Former	9 (17.0)
Never	35 (67.0)
**Lung function**	
FVC_pred., median (IQR), %	94 (85, 104)
FEV1_pred., mean (SD), %	92 (10.5)
FEV1/FVC, mean (SD)	79 (7.7)
TLco pred., mean (SD), %	83 (15.8)
Kco pred., mean (SD), %	91 (18.7)
**Reduced Employment due to Long COVID-19, N (%)**	22 (7.8)
Reduced ≥50%	10 (14.5)
Reduced <50%	12 (17.4)
**Pre-existing comorbidities, N (%)**	
Asthma	7 (13.0)
COPD	1 (2.0)
Hypertension	6 (12.0)
Cardiovascular diseases	2 (4.0)
Psychiatric disorders (depression, anxiety, and adaption disorder)	11 (21.0)

SD: standard deviation; IQR: interquartile range; BMI: body mass index; WHO: World Health Organization; CT: computer tomography; FVC: forced vital capacity; pred.: predicted; FEV1: forced expiratory volume in 1 s; TLco: diffusion capacity of the lung for carbon monoxide; Kco: carbon monoxide transfer coefficient; COPD: chronic obstructive pulmonary disease.

**Table 2:** Chest CT scan findings (N = 53).

Possible post-COVID-19 features	29 (54.7)
Mosaik attenuation	21 (39.6)
Fibrotic like changes	13 (24.5)
Parenchymal bands	4 (7.5)
Reticulation	9 (17.0)
Mediastinal and bihilar lymphadenopathy	1 (1.9)
Consolidation	1 (1.9)
Nodules	1 (1.9)
Band-like consolidation	1 (1.9)
Ground glass opacity	11 (20.8)
Emphysema	6 (11.3)
Honey combing	1 (1.9)

Values are presented as N (%).

**Table 3:** Inter-rater reliability of chest CT scan findings.

	Rater 1	Rater 2	Agreement (%)	Cohen’s Kappa (k)
Post-COVID-19 features	30 (56.6)	31 (58.5)	94.3	0.88
Mosaic attenuation	21 (39.6)	22 (41.5)	98.1	0.96
Fibrotic-like changes	14 (26.4)	14 (26.4)	96.2	0.90
Ground glass opacity	10 (18.9)	12 (22.6)	96.2	0.89
Emphysema	6 (11.3)	7 (13.2)	98.1	0.91
Honey combing	1 (1.9)	1 (1.9)	100	1.00

Values are presented as N (%) unless otherwise stated.

**Table 4:** Multivariable regression models between chest CT scan findings and persisting symptoms.

Thoracic Pain	Odds Ratio (95% CI)	*p*-Value
Post-COVID-19 features	0.42 (0.13, 1.39)	0.154
Fibrotic-like changes	0.91 (0.24, 3.43)	0.894
Ground glass opacity	0.61 (0.16, 2.36)	0.473
Mosaic attenuation	0.44 (0.14, 1.42)	0.169
**Dyspnea**		
Post-COVID-19 features	1.02 (0.20, 5.19)	0.981
Fibrotic-like changes	7.48 (0.57, 97.65)	0.125
Ground glass opacity	4.47 (0.34, 58.38)	0.253
Mosaic attenuation	0.70 (0.15, 3.32)	0.655
FEV1 % pred.	1.05 (0.97, 1.06)	0.537
Kco % pred.	0.93 (0.83, 1.04)	0.200
**Cough**		
Post-COVID-19 features	0.65 (0.21, 2.01)	0.453
Fibrotic-like changes	1.16 (0.32, 4.23)	0.818
Ground glass opacity	0.74 (0.18, 2.92)	0.854
Mosaic attenuation	0.50 (0.16, 1.63)	0.252

CT: computed tomography; FEV1 %: forced expiratory volume in 1 s; pred.: predicted; Kco: carbon monoxide transfer factor.

## Data Availability

The datasets generated and analyzed during the current study are not publicly available but are available from the corresponding author on reasonable request.

## Supplementary Materials

The following supporting information can be downloaded at https://www.mdpi.com/article/10.3390/diagnostics13091616/s1: Table S1. Current symptoms after SARS-CoV-2 infection.
