# Supplementary material for: Chest CT Findings after Mild COVID-19 Do Not Explain Persisting Respiratory Symptoms: An Explanatory Study
Source: Diagnostics (Basel). 2023 May 3;13(9):1616. doi: 10.3390/diagnostics13091616 (PMC10178158; doi:10.3390/diagnostics13091616)
Supplement: Supplementary file 1 [file diagnostics-13-01616-s001.zip › diagnostics-2329298-supplementary.pdf]

diagnostics

Article

Chest CT findings after mild COVID-19 do not explain persisting respiratory symptoms: an explanatory study

Supplementary material

Stefan Malesevic [1,2,†], Noriane A. Sievi [2,†], Jonas Herth [1,2], Felix Schmidt [1,2], Dörthe Schmidt [3], Florence Vallelian [4], Ilijas Jelcic [5], Lisa Jungblut [6], Thomas Frauenfelder [1,6], Malcolm Kohler [1,2], Katharina Martini [6] and Christian F. Clarenbach [1,2,*]

[1] Faculty of Medicine, University of Zurich, 8006 Zurich, Switzerland
[2] Department of Pulmonology, University Hospital Zurich, 8091 Zurich, Switzerland
[3] Department of Cardiology, University Hospital Zurich, 8091 Zurich, Switzerland
[4] Department of Internal Medicine, University Hospital Zurich, 8091 Zurich, Switzerland
[5] Department of Neurology, University Hospital Zurich, 8091 Zurich, Switzerland
[6] Institute of Diagnostic and Interventional Radiology, University Hospital Zurich, 8091 Zurich, Switzerland

Citation: Malesevic, S.; Sievi, N.A.; Herth, J.; Schmidt, F.; Schmidt, D.; Vallelian, F.; Jelcic, I.; Jungblut, L.; Frauenfelder, T.; Kohler, M.; et al. Chest CT Findings after Mild COVID-19 Do not Explain Persisting Respiratory Symptoms: An Explanatory Study. *Diagnostics* **2023**, *12*, x. https://doi.org/10.3390/xxxxx

Academic Editor(s): Fabiano Bini; Mariano Scaglione

Received: 22 March 2023
Revised: 24 April 2023
Accepted: 28 April 2023
Published: date

Copyright: © 2023 by the authors. Submitted for possible open access publication under the terms and conditions of the Creative Commons Attribution (CC BY) license (https://creativecommons.org/licenses/by/4.0/).

Table S1. Current symptoms after SARS-CoV-2 infection.

High temperature (N=37)	6 (16.2)
Cough (N=49)	22 (41.5)
Orthopnea (N=24)	0 (0)
Dyspnea (N=53)	42 (79.3)
mMRC=1	31 (73.8)
mMRC=2	7 (16.7)
mMRC=3	3 (7.1)
mMRC=4	1 (2.4)
Sputum (N=43)	12 (27.9)
Muscle pain (N=39)	16 (41.0)
Joint pain (N=9)	5 (55.6)
Thoracic pain (N=53)	34 (64.2)
Palpitations (N=40)	11 (27.5)
Muscle weakness (N=37)	16 (43.2)
Raynaud syndrome (N=35)	0 (0)
Swallowing (N=34)	3 (8.8)
Reflux (N=37)	5 (13.5)
Fatigue (N=48)	45 (93.8)
Dizziness (N=38)	13 (34.2)
Concentration weakness (N=40)	32 (80.0)
Memory loss (N=13)	11 (84.6)
Tingling paresthesia (N=3)	1 (33.3)
Weight gain (N=41)	7 (17.1)
Weight loss (N=42)	14 (33.3)
Smell alteration (N=43)	19 (44.2)
Taste alteration (N=41)	17 (41.5)
Sleep disturbance (N=41)	16 (39.0)
Headache (N=37)	15 (40.5)
Hair loss (N=1)	1 (100)
Performance intolerance (N=29)	28 (96.6)
Post-exertional malaise (N=7)	6 (85.7)
Gastrointestinal disorders (N=41)	7 (16.3)

Values are presented as N (%). Only patients with a recorded "present/absent" in the medical records were included. mMRC: modified medical research counceling dyspnea scale.
